# Association between hyponatremia and adverse clinical outcomes of heart failure: current evidence based on a systematic review and meta-analysis

**DOI:** 10.3389/fcvm.2023.1339203

**Published:** 2023-12-22

**Authors:** Wanling Zhao, Jiangwei Qin, Guoyan Lu, Yang Wang, Lina Qiao, Yifei Li

**Affiliations:** Department of Pediatrics, Ministry of Education Key Laboratory of Women and Children’s Diseases and Birth Defects, West China Second University Hospital, Sichuan University, Chengdu, Sichuan, China

**Keywords:** hyponatremia, heart failure, adverse events, mortality, meta-analysis

## Abstract

**Background:**

Heart failure (HF) is a global health challenge. The perturbations in fluid and electrolyte equilibrium, particularly the compromised sodium balance associated with HF lead to high mortality rates. Hence, elucidating the correlation between serum sodium levels and the prognosis of HF is of paramount importance. This study aimed to conduct a comprehensive meta-analysis to thoroughly investigate the interplay between hyponatremia and the prognostic outlook of individuals with HF.

**Methods:**

A comprehensive search of bibliographic databases including PubMed, Embase, and the Cochrane Central Register of Controlled Trials was conducted to identify relevant observational studies examining the association between hyponatremia and prognosis of HF. Data extraction, synthesis, and assessment of risk of bias were conducted. Meta-analytic methods, sensitivity analyses, and heterogeneity test were employed as appropriate to synthesize the data.

**Results:**

A total of 43,316 patients with HF were included spanning 25 selected studies. The pooled data revealed a notable association between hyponatremia and elevated risks across short and long-term mortality of HF. Specifically, hyponatremia was found to significantly increase the likelihood of all-cause mortality (Hazard ratio [HR] = 1.94, 95% confidence interval [CI]: 1.78–2.12); 1-year mortality (HR = 1.67, 95%CI: 1.46–1.90); 30-day mortality (HR = 2.03, 95%CI: 1.73–2.25); cardiac mortality (HR = 2.11, 95%CI: 1.81–2.46); and in-hospital mortality (HR = 1.64, 95%CI: 1.15–2.34).

**Conclusion:**

Our meta-analysis emphasizes the significant impact of hyponatremia on mortality in the HF patient population, highlighting the critical importance of maintaining stable serum sodium levels in HF management.

## Introduction

Heart failure (HF) represents a global epidemic linked to significant mortality rates and substantial economic burden. Its prevalence has steadily risen owing to improved survival rates of cardiac arrest due to coronary artery diseases and an aging population with failing heart, accounting for 1%–3% of the adult population in developed countries. Currently, an estimated 37.7 million cases have been reported annually, resulting in 4.2 million years of compromised health (1, 2).

Heart failure-related electrolyte balance damage may involve the following physio-pathological processes: The decreased cardiac output and poor circulation can impact kidney function, leading to reduced filtration and fluid reabsorption. Furthermore, advanced HF increases the risk of arrhythmias due to electrical instability and changes in the heart's structure (3–5). Heart failure triggers the activation of the renin-angiotensin-aldosterone system (RAAS) and the sympathetic nervous system (SNS). These pathways contribute to sodium and water retention, as well as vascular constriction, exacerbating fluid overload. The elevated levels of arginine vasopressin (AVP) in HF lead to increased water reabsorption in the renal collecting ducts via V2 receptors, further compounding fluid retention. Moreover, altered endothelial function also plays a role leading to increased capillary permeability and impaired sodium transport mechanisms. Furthermore, chronic inflammation in HF promotes sodium and water retention by modulating sodium channels and transporters, such as the epithelial sodium channel (ENaC) and sodium-potassium pump (Na^+^/K^+^-ATPase). Other types of ion channels including potassium and calcium channels are also frequently disturbed in HF.

Among the various types of electrolyte-homeostasis disorders associated with HF, hyponatremia emerges as a common consequence due to abnormal fluid and electrolyte regulation. This results in volume overload-induced dilutional hypervolemic hyponatremia as mentioned, as well as hypovolemic hyponatremia arising from the excessive use of natriuretics such as thiazide diuretics. In patients with HF, the prevalence of hyponatremia ranges between 11% and 27%, imparting a two-fold increase in mortality risk (5, 6). Some meta-analyses have substantiated that hyponatremia serves as a potent predictor of mortality in HF patients, irrespective of ejection fraction (7). Moreover, enhanced sodium levels are reportedly associated with reduced mortality risk, although these conclusions are based on a restricted pool of samples and varying HF subtypes (8). The prevailing therapeutic approaches include AVP V2 receptor antagonists that target the predominant form of mammalian antidiuretic hormone (ADH), such as tolvaptan (TLV) or satavaptan (STV). Fluid restriction and non-specific strategies including diuretic therapy for congestion and administration of isotonic or hypertonic saline are also employed (5, 9). However, despite these interventions, the optimal management strategy for hyponatremia in HF patients remains an unresolved issue (10). Thus, it is critical to evaluate and understand the association between serum sodium level and HF prognosis, which would aid in prognosis of HF and therapeutic targets.

Hence, this study aimed to perform a comprehensive meta-analysis to evaluate the link between hyponatremia and adverse prognosis in patients with HF. Additionally, we conducted a systematic review of randomized controlled trials (RCTs) that focused on the various treatment modalities for managing HF complicated by hyponatremia.

## Methods

This systematic review protocol adheres to the guidelines outlined in the Preferred Reporting Items for Systematic Reviews and Meta-Analysis (PRISMA) statement. The protocol for this review has been registered in the PROSPERO International Prospective Register of systematic reviews (CRD42022323116). As this study involves a systematic literature research, the need for ethical approval was waived (11).

### Eligibility criteria

The inclusion criteria were as follows: (1) the study should involve HF patients without causes mentioned in the exclusion criteria. Both preserved and reduced ejection fraction cases were considered; (2) the study design should be a prospective or retrospective observational study; (3) the standard for identifying hyponatremia should be addressed; (4) clinical outcomes should be included, such as all-cause mortality, or other short-term and long-term adverse events; (5) all the results associated with clinical outcomes should be presented as hazard ratio (HR) or data which could be calculated as HR for hyponatremia. The exclusion criteria were as follows: (1) all animal studies, case reports, and case series analyses; (2) the HF was due to critical congenital heart diseases with or without surgical correction; (3) any cardiac syndrome/abnormality induced by chromosomal or genetic disorders; (4) patients with suspected myocarditis; (5) patients who have undergone heart transplantation or received a left ventricular assist device.

### Search strategy

From inception to 31 March 2023, PubMed, Embase and the Cochrane Central Register of Controlled Trials had been searched. Searching strategy is as followed (example in Pubmed): (“hyponatraemia"[All Fields] OR “hyponatremia"[MeSH Terms] OR “hyponatremia"[All Fields] OR “hyponatremias"[All Fields] OR ((“serum"[MeSH Terms] OR “serum"[All Fields] OR “serums"[All Fields] OR “serum s"[All Fields] OR “serumal"[All Fields]) AND (“sodium"[Supplementary Concept] OR “sodium"[All Fields] OR “sodium"[MeSH Terms] OR “sodiums"[All Fields])) OR ((“serum"[MeSH Terms] OR “serum"[All Fields] OR “serums"[All Fields] OR “serum s"[All Fields] OR “serumal"[All Fields]) AND “natrium"[All Fields])) AND (“heart failure"[MeSH Terms] OR (“heart"[All Fields] AND “failure"[All Fields]) OR “heart failure"[All Fields]).

### Study selection and quality assessment

Citations were collected using a reference manager software program (EndNote 22.0) and duplicates were eliminated. Two authors (WZ and JQ) selected studies by assessing the titles and abstracts after excluding the duplicate entries. The full text was further reviewed for inclusion. Disagreements were resolved by discussion with a third author (YL), until consensus was reached. We also recorded reasons for exclusion of studies. For assessment of cohorts, the Newcastle–Ottawa Scale (NOS) with eight items was used to assess the quality of the cohort studies. One or two points were awarded for each criterion, and the points were added up to compare study quality in a quantitative manner. Total points of <5 and ≥5 were assigned for unacceptable and acceptable quality of studies, respectively. Two reviewers independently carried out the assessments.

### Data extraction and analysis

A standard data collection was performed across the included studies. Two reviewers (WZ and JQ) independently extracted the data from the included studies and filled out the data collection form. The following data were extracted and organized:

• General information: First author; Article title; Publication journal; Year of publication; Countries the major population enrolled;
• Methods: Study design; Control setting; Whether randomization was involved; Distribution concealment method; Blinding method; Inclusion and exclusion criteria of the enrolled population; Echocardiographic assessment; Cardiac MRI measurement; Family history;
• Subjects: Age; Sex; Severity of HF (AHA stage and NYHA classification); Time duration since HF diagnosis; Results of genetic analysis; Ethnicity; Criteria for the diagnosis of hyponatremia and definition of improvement of hyponatremia; Other complications
• Intervention: Medication or device used for HF treatment;
• Results: Follow-up period; Survival rate; Cardiac function improvement; All-cause mortality; In-hospital mortality, 1-month mortality; 1-year mortality.

### Statistical analyses

Stata 15.0 software was used for all data analyses and synthesis in this meta-analysis. The outcomes associated with hyponatremia were assessed as hazard ratios (HRs) with 95% confidence intervals (CIs). *I*^2^ was used to evaluate the heterogeneity, wherein, *I*^2 ^> 50% was considered to indicate significant heterogeneity, and the random-effects model was chosen. Sensitivity analysis was performed to test the robustness of the results using the leave-one-out analysis and meta-regression analysis. In all groups, a leave-one-out analysis was conducted, while meta-regression was exclusively applied to the all-cause mortality group owing to unavailability of covariate data, which could potentially introduce bias into the outcomes. Potential publication bias was assessed by funnel plots with the Egger's regression asymmetry test. Meta-regression was executed using the R package. Image enhancement procedures were implemented through Graphpad Prism.

## Results

**Figure 1:**
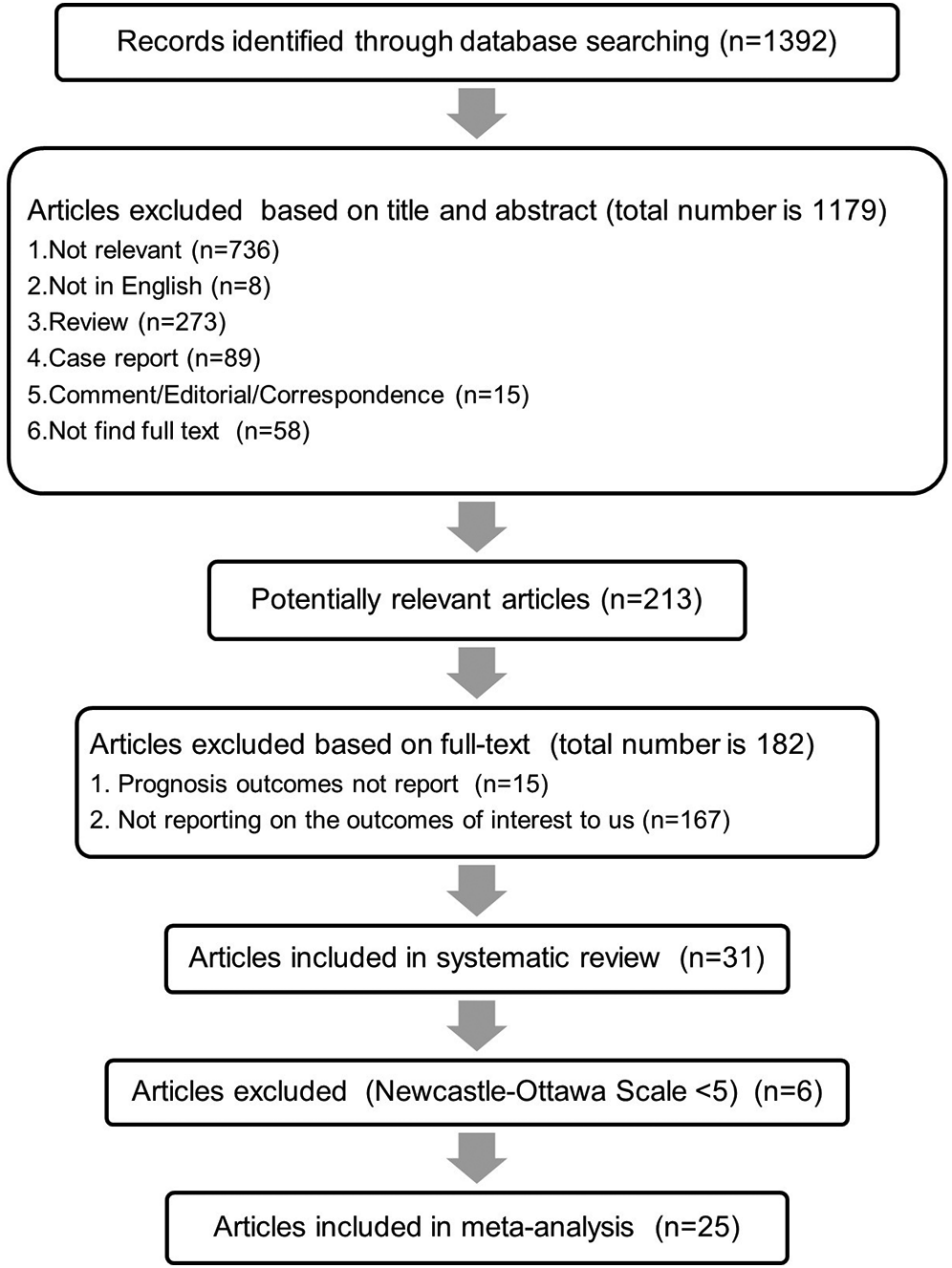
The processes of database search and study selection.

The methodology for database search and article screening is presented in Figure 1. Initially, a total of 1,392 articles were identified through the database search. Subsequent screening of titles and abstracts resulted in the exclusion of 1,179 articles. The remaining 213 records were evaluated in detail for eligibility through full-text review. Among these, 182 articles were excluded for their lack of relevance to our defined outcomes of interest. Following the assessment of study quality, six articles were excluded based on the NOS evaluation. Ultimately, a total of 25 articles, meeting the criterion of NOS scores >5, were deemed suitable for inclusion in the final analysis.

The cohort characteristics of the included studies are summarized in Table 1. A total of 43,316 patients with HF were included across 25 selected studies (12–38), spanning the years 2003–2023. The mean age of patients ranged from 58.7 to 78.4, with males constituting 42.2%–100% among the different cohorts. Notably, comorbidities such as hypertension, diabetes, and COPD were prevalent among the HF patients. Approximately 37–60.66% patients were classified under NYHA classes III–IV, indicating advanced stages of HF. Among the 25 included articles encompassing 43,316 patients, data on serum sodium concentration were available in 18 studies. The threshold value for diagnosing hyponatremia was mainly set as 135 mmol/L, with one study setting it at 136 mmol/L and another at 140 mmol/L. The incidence of hyponatremia ranged widely, spanning 5%–50%. The follow-up duration exhibited considerable variability, ranging from the duration of hospitalization to 7 years post-discharge. Of the eligible studies, five reported 1-year mortality, 14 reported all-cause mortality, four considered 30-day mortality, four assessed cardiac mortality, and another three studies accounted for in-hospital mortality as the outcome measure.

**Table 1:** The characteristics of the included cohort studies.

Author	Year of publication	Na^+^ cut-off threshold (mEq/L)	Follow-up (year)	Sample size	HF phenotype	Age (years)	Gender, male (%)	Hypertension (%)	Diabetes (%)	COPD (%)	NYHA III (%)	NYHA IV (%)	NYHA III–IV (%)	Na ^+ ^(mmol/L) (mean ± SD)	Hyponatremia (%)	NOS
Lee, D. S.	2003	136	NA	2,624	HF	76.3	49.5	NA	34	20.7	NA	NA	NA	138	NA	5
Senni, M.	2005	135	1	1,315	CHF	64 + 12	78	NA	NA	NA	NA	NA	37	140 + 4	5	5
Jafary, F. H.	2007	135	1.04	196	SHF	61.2	64.8	67.3	60.7	NA	NA	NA	NA	NA	50	5
Vazquez, R.	2009	138	3.7	992	CHF	65 + 12	72.4	57	35.9	NA	21.6	NA	NA	NA	37.7	7
Rusinaru, D.	2009	136	7	358	HFpEF	76 ± 10	47	NA	NA	NA	NA	NA	NA	138	25.4	5
Spinar, Jindrich	2011	130	NA	4,153	AHF	71.5 ± 12.4	47.6	73.1	42.6	16.2	NA	NA	43.2	139	5	5
Felšöci, M.	2011	130	NA	2,421	AHF	73.6	NA	71.54	40.79	17.02	NA	NA	52.71	NA	NA	6
Bettari, L.	2012	135	4.5	1,045	HF	62	67	67.4	35	NA	42.4	29.4	NA	NA	10.2	5
Oh, C.	2012	135	0.2 ± 1.49	239	HF with left ventricular ejection fraction ≤35% and QRS interval >120 ms	67 ± 11	66.95	55	39	NA	NA	NA	NA	NA	NA	5
Kozdag, G.	2013	NA	NA	409	CHF	64 ± 12	100	70	34	NA	NA	NA	NA	shown in subgroup	5.13	5
Sato, N.	2013	135	NA	4,837	HF	73.0 ± 13.8	57.9	69.3	33.8	NA	37.8	43.6	NA	139.3 ± 4.4	NA	5
Carrasco-Sánchez, F. J.	2014	135	1	195	AHF	76.3 ± 8.2	42.2	81.1	48.2	31.2	NA	NA	37.4	137 ± 4.9	23	6
Carrasco-Sánchez, F. J.	2014	135	1	1,082	HF	77.61	45.1	86	45.29	27.7	38.4	3.4	NA	138.8	NA	6
Carlo, C. H.	2014	NA	NA	333	SHF	58.7 ± 15.4	64	NA	NA	NA	NA	NA	NA	136.6 ± 4.9	NA	5
Herrero-Puente, P.	2014	135	NA	4,700	AHF	NA	NA	NA	NA	NA	NA	NA	NA	NA	NA	6
Coles, A. H.	2015	135	5	4,025	35% HFrEF 13% HF HFmrEF 52%HFpEF	75	44	NA	NA	NA	NA	NA	NA	shown in subgroup	NA	6
Kapłon-Cieślicka, A.	2015	135	NA	641	HF	69	64	NA	NA	NA	NA	NA	NA	138.4	15.8	6
Kajimoto, K.	2016	136	1.42	4,438	AHF	NA	NA	NA	NA	NA	NA	NA	NA	shown in subgroup	NA	6
Kajimoto, K.	2016	135	1.44	4,628	AHF	72.8 ± 13.8	58.1	69.4	33.6	12.3	38	43.7	NA	139.3 ± 4.4	NA	7
Yoshioka, K.	2016	135	NA	882	AHF	NA	NA	NA	NA	NA	NA	NA	NA	shown in subgroup	NA	5
Formiga, F.	2018	135	1	985	AHF	78.4 ± 9	45.3	NA	NA	NA	NA	NA	NA	138 ± 4	15.2	6
Givi, M.	2018	135	NA	1,223	HF	NA	NA	NA	NA	NA	NA	NA	NA	NA	NA	6
Sato, Y.	2019	135	NA	500	HFpEF	NA	NA	NA	NA	NA	NA	NA	NA	shown in subgroup	NA	6
Alem, M. M.	2020	135	NA	241	CHF	60.61 ± 12.63	65.1	77.18	71.37	10.79	32.19	15.45	NA	138	14.11	5
Yang, M.	2020	140	NA	854	HF	76.72 ± 6.40	56.21	79.27	39.58	NA	NA	NA	60.66	139.56 ± 4.74	NA	5

NA, not available; AHF, acute heart failure; CHF, chronic heart failure; HFrEF, heart failure with reduced ejection fraction; HFpEF, heart failure with preserved ejection fraction; HFmrEF, heart failure with mid-range ejection fraction; NYHA, New York Heart Association classification; NOS, Newcastle-Ottawa Scale.

### Analysis of the association between hyponatremia and mortality

**Figure 2:**
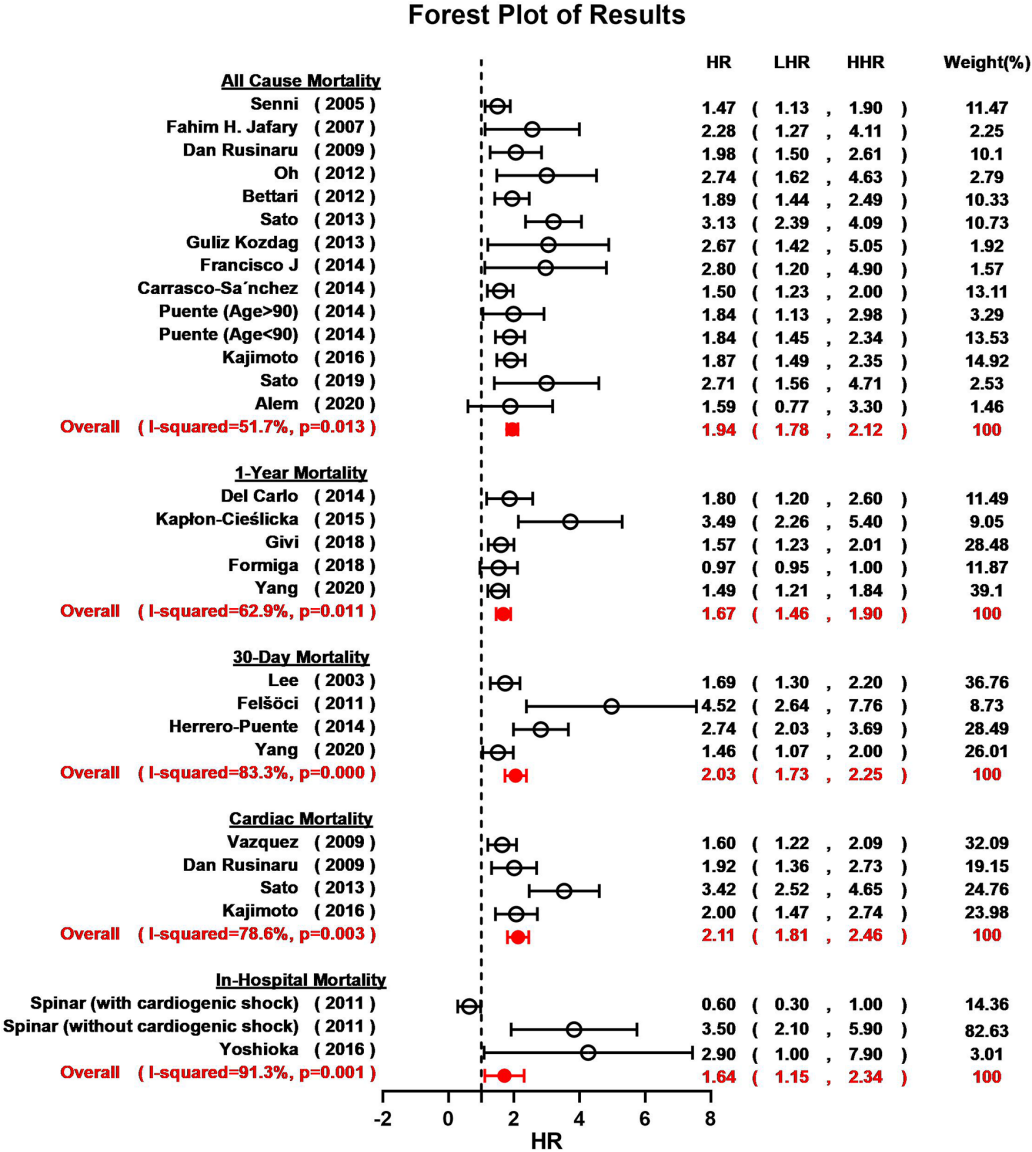
Forest plot for the meta-analysis regarding the relationship between hyponatremia and all-cause mortality, 1-year mortality, 30-day mortality, cardiac mortality and in-hospital mortality risk in patients HF and hyponatremia.

**Figure 3:**
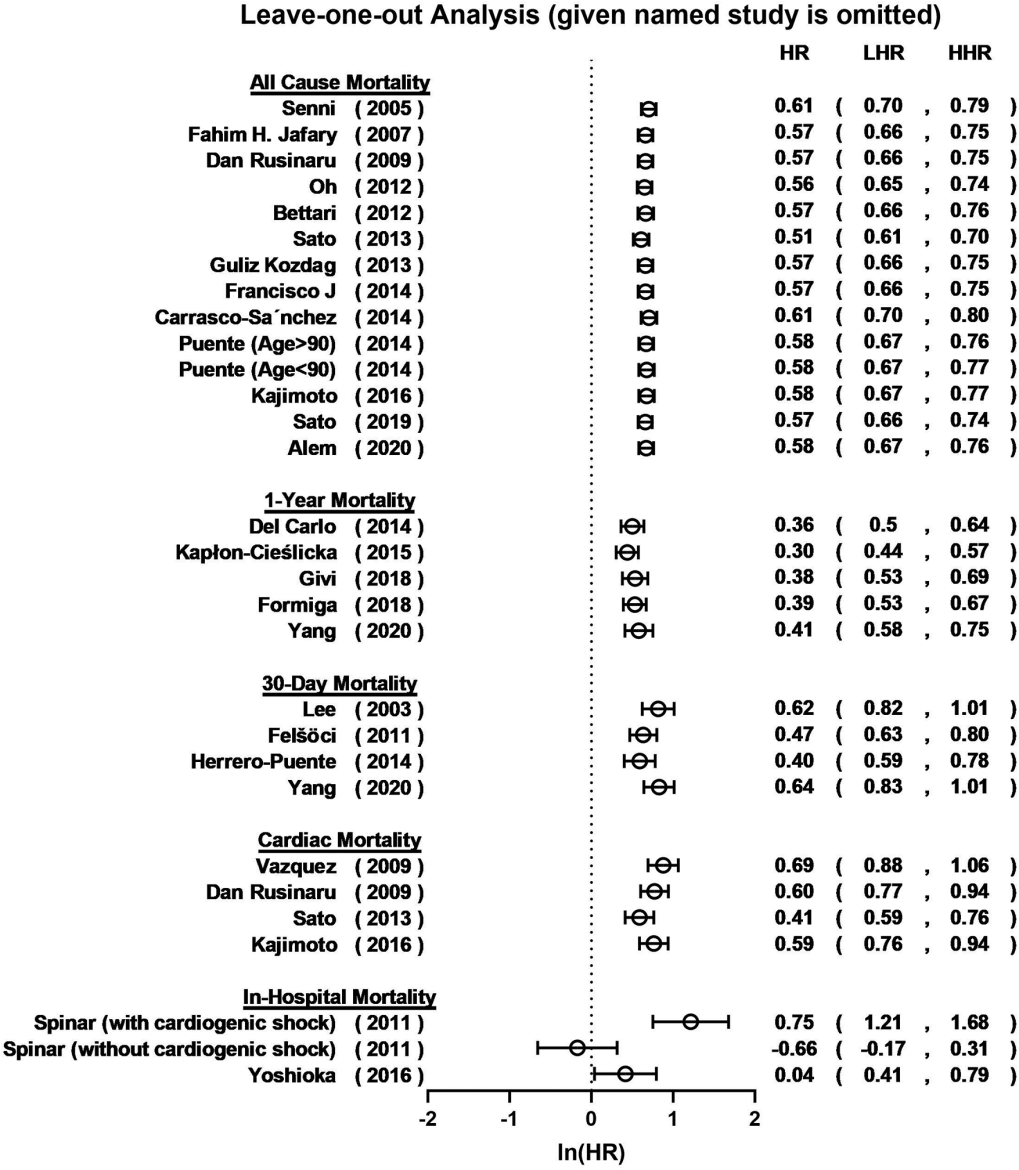
Leave-one-out plot for sensitivity analysis of the relationship between hyponatremia and all-cause mortality, 1-year mortality, 30-day mortality, cardiac mortality and in-hospital mortality risk in patients HF and hyponatremia.

**Figure 4:**
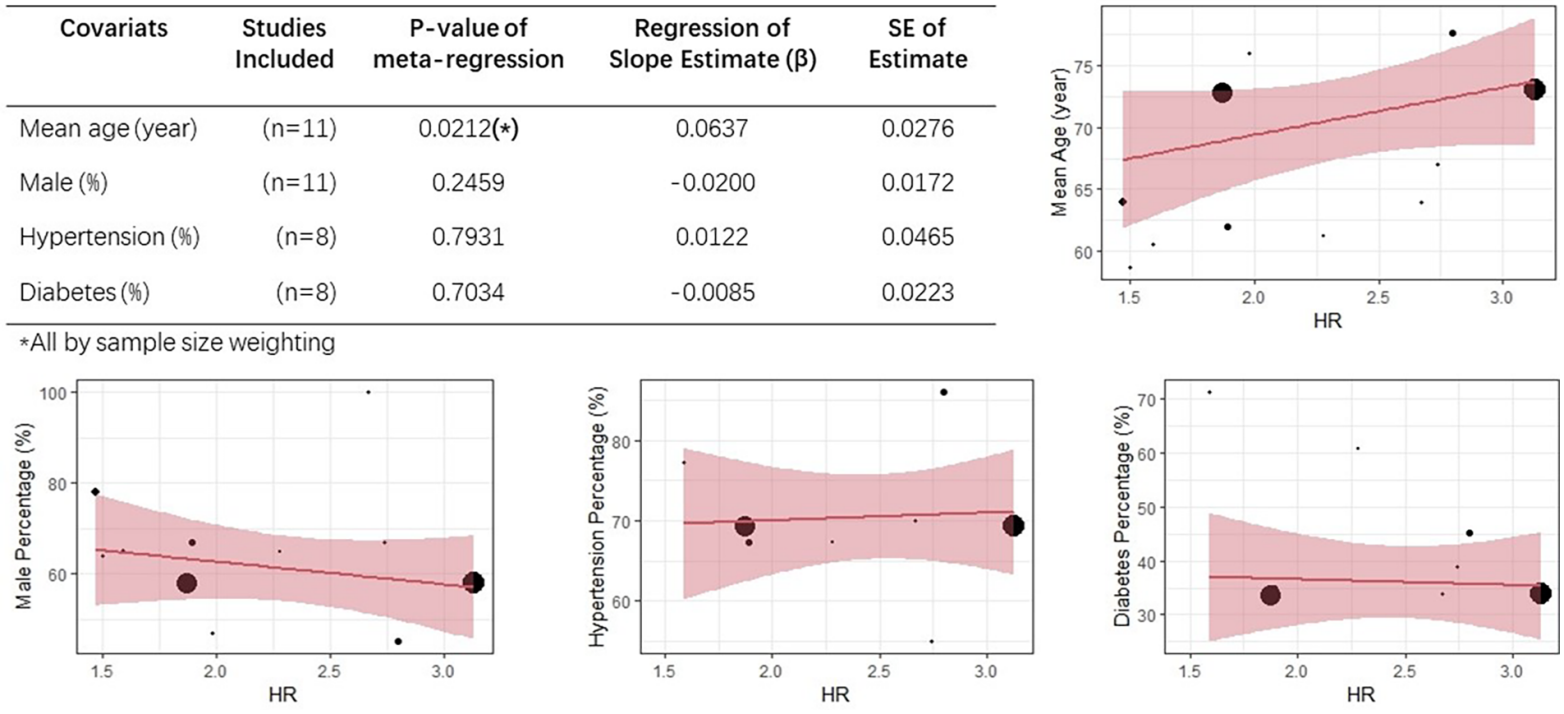
Meta-regression analyses based on mean age, male percentage, hypertension percentage, diabetes percentage.

**Figure 5:**
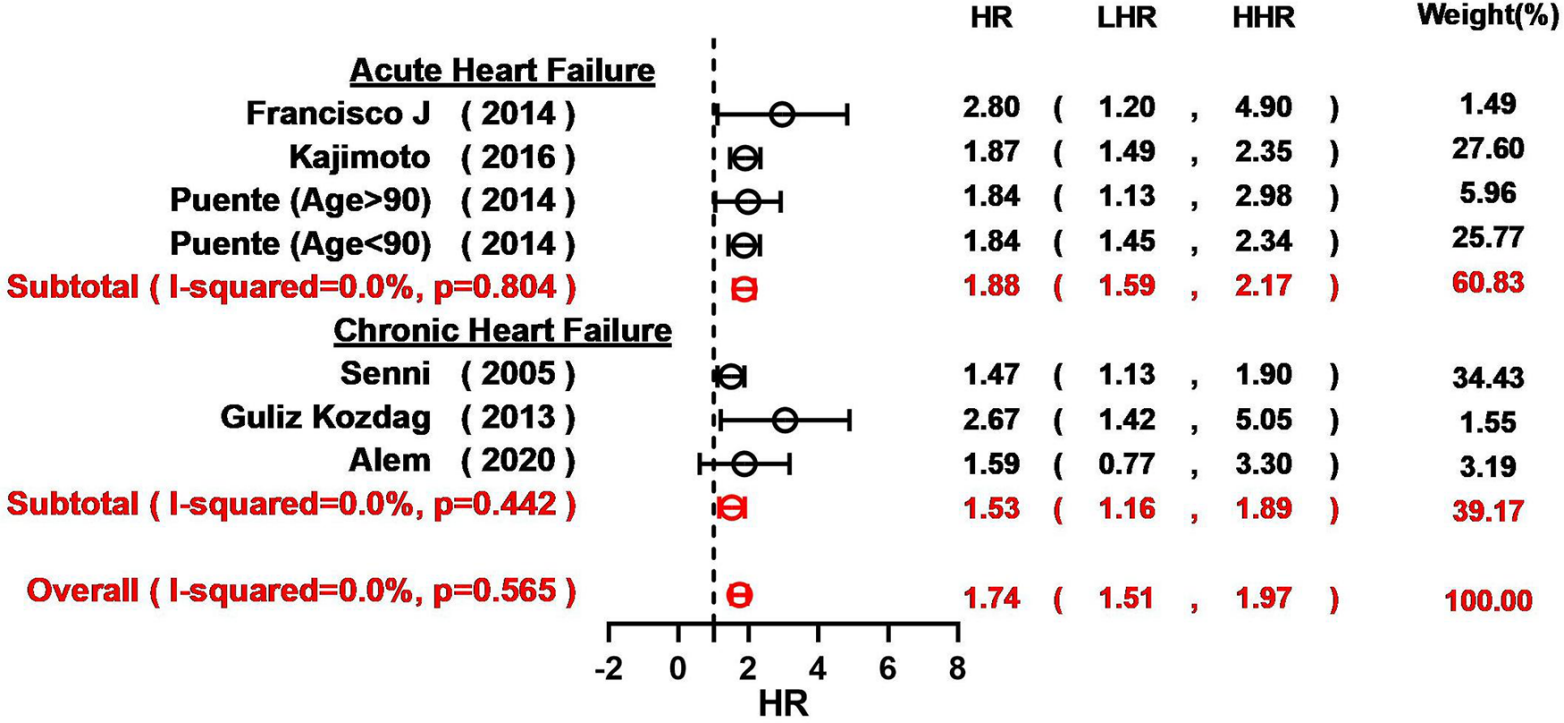
Forest plot for the meta-analysis based on HF phenotype.

Overall, hyponatremia exhibited a significant association with an elevated risk of all-cause mortality in patients with HF (HR = 1.94, 95% CI: 1.78–2.12; *I*^2 ^= 51.7%; Figure 2). Furthermore, a notable increase in the 1-year mortality was evident among HF patients with hyponatremia (HR = 1.67, 95% CI: 1.46–1.90; *I*^2 ^= 69.2%; Figure 2). The odds of 30-day mortality were two-fold higher (HR = 2.03, 95%CI: 1.73–2.25; *I*^2 ^= 83.8%; Figure 2). A nearly doubled risk of cardiac mortality was also observed (HR = 2.11, 95% CI: 1.81–2.46; *I*^2 ^= 78.6%; Figure 2). And there was also an increased risk of in-hospital mortality in HF patients associated with hyponatremia compared to non-hyponatremia (HR = 1.64, 95% CI: 1.15–2.34; *I*^2 ^= 91.3%; Figure 2). Given the considerable heterogeneity across the studies, the random-effects model was employed to compute pooled HR estimates. The results from the included studies consistently indicated that hyponatremia was linked to a heightened risk of mortality. A leave-one-out analysis that excludes studies one-by-one, indicated a consistent and robust tendency in the direction and magnitude of association (Figure 3). Meta-regression analyses identified that mean age of patients may influence the meta-synthesis (*p* = 0.0177), while the scatter plot indicated increased all-cause mortality with elevated mean age of patients. Nonetheless, the most diminutive age cohort, with an average age of 58.7 ± 15.4, still manifested a heightened susceptibility to hyponatremia (HR = 1.80, 95% CI: 1.60–2.60). We also conducted meta-regression based on male percentage (*p* = 0.2459), complications percentage including hypertension (*p* = 0.7931) and diabetes (*p*-value = 0.7034), which all indicated a minimal influence on the raw results (Figure 4). The preceding analysis demonstrate a relatively stable correlation between the elevated lethality and hyponatremia in HF patients aged ≥50 years. Furthermore, the subgroup analysis yielded compelling evidence indicating a significant disparity in all-cause mortality between the acute heart failure (AHF) phenotype and the chronic heart failure (CHF) phenotype (Figure 5), suggesting higher mortality in AHF than that in CHF.

### Quality assessment and publication bias

**Figure 6:**
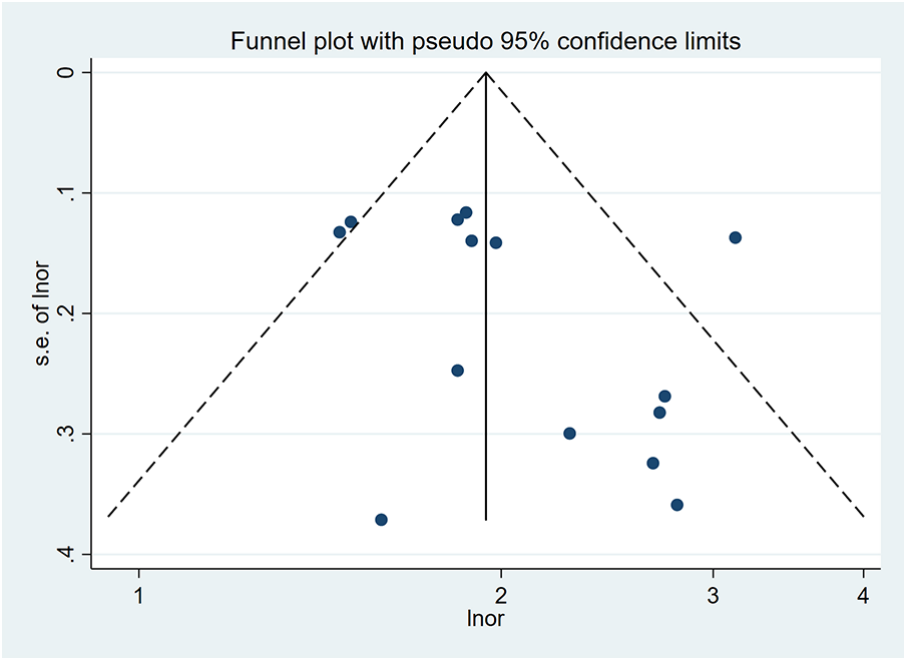
Funnel plot of all-cause mortality.

Quality assessment was performed using the NOS, revealing scores ranging from 5 to 7 across the included studies. Detailed scores of three parts of NOS (selection, comparability, exposure) were presented in Supplementary Table S1. The funnel plot for the all-cause mortality group is presented in Figure 6. Moreover, Egger's test demonstrated the absence of publication bias in all groups, including all-cause mortality (*p* = 0.199), 1-year mortality (*p* = 0.218), 30-day mortality (*p* = 0.301), cardiac mortality (*p* = 0.696), and in-hospital mortality (*p* = 0.496).

## Discussion

In this comprehensive meta-analysis, we systematically included a substantial cohort of 43,316 patients from 25 published studies, encompassing diverse age groups, mainly of older individuals. Consistent with previous research, our study underscores a robust association between hyponatremia and a significantly compromised prognosis among a wide spectrum of HF patients. Specifically, our findings reveal a substantial 1.5-time increase in the risk of 1-year mortality and in-hospital mortality, and a noteworthy nearly double elevation in all-cause mortality, 30-day mortality and cardiac mortality. Age and HF phenotypes were significant covariates.

The pathogenesis of hyponatremia in patients with HF is multifaceted. Within the context of HF, compromised cardiac function leads to insufficient effective intravascular volume and venous congestion. Traditionally, the management of water retention has heavily relied on diuretics, many of which exacerbate electrolyte disturbances, including hyponatremia. The incidence of hyponatremia is notably substantial among patients with HF, often ranging between 11% and 27% (5, 6). In more severe clinical contexts, this prevalence could encompass up to half of all HF individuals (39). A nested case-control investigation within the Korean Acute Heart Failure registry, comprising 2,935 patients, revealed that severe and persistent hyponatremia amplified the risk of death by nearly 90% and escalated the likelihood of right ventricular dysfunction by over eight-fold in patients with acute HF (40). In cases of HF with concomitant hyponatremia (HFcHN), the hypotonic state can foster intracellular water accumulation, precipitating cellular edema (41). The severity of hyponatremia can result in acute water intoxication, which prompts edema in brain tissue, triggers diverse psychiatric symptoms, and in extreme scenarios, precipitates severe herniation (42). Swift and systematic rectification of serum sodium levels is crucial to avert acute water intoxication. Timely restoration of serum sodium levels has been demonstrated to effectively curtail all-cause mortality. A meta-analysis conducted by Jinhui et al. highlighted that timely hyponatremia correction in patients with HFcHN was correlated with a reduced risk of mortality, particularly short-term mortality (8). Several studies have consistently demonstrated a positive correlation between the amelioration of hyponatremia and improved prognosis among patients with HF (32, 43, 44). Conversely, untreated or severe hyponatremia (<130 mmol/L) has been associated with heightened mortality rates and inferior outcomes (32, 33, 37, 45, 46).

Potassium and magnesium homeostasis also demonstrates great significance in the management of HF patients. Low potassium serum levels promote the development of hyponatremia by shifting sodium into the cell and cause higher mortality of arrhythmic death (47). Potassium disturbance is a potent predictor both in short-term and long-term mortality of HF patients (48). Magnesium are involved in the normal function of Na^+^/K^+^-ATPase, thus, hypomagnesemia can also lead to lower extracellular sodium levels and ischemic stroke, and cardiac arrhythmias can be prevented or treated with the improvement of magnesium (49–51).

Notably, while the commonly employed threshold for high-risk hyponatremia in HF is defined as a serum sodium level below 135 mmol/L, the optimal range of serum sodium concentration for predicting an adverse prognosis in HF may be higher (52). Notably, previous studies have proposed a linear relationship between mortality, rehospitalization, and serum sodium concentrations <140 mmol/L, which includes the conventional reference limits of 135–140 mmol/L (7, 28, 53). The risk of death or rehospitalization increased by 8% for each 3 mmol/L decrease in admission serum sodium below 140 mmol/L (53). Deubner et al. even proposed a U-shaped correlation between serum sodium concentration and mortality risk, designating the optimal range as high normal sodium levels (130–139 mmol/L) (54). Among patients with persistent hyponatremia, those with more severe hyponatremia at discharge (<130 mmol/L) were proved to have higher 30day readmission or mortality, than those with less severe hyponatremia at discharge (130–134 mmol/L) (46). Moreover, for the management of the HF patient complicated hyponatremia, saline administration or fluid restriction and the use of hypertonic saline with loop diuretics are main treatments in dilutional or depletiona phenotypes of hyponatremia (47). Close monitoring and appropriate protocol of serum sodium correction are employed to avoid rapid correction, especially in severe hyponatremia (<125 mmol/L) (41, 42). In summary, the most favorable range of Na^+^ should be 140–145 mmol/L. Nevertheless, it is quite unrealistic considering clinical situation. In our scholarly perspective, it is recommended that sodium level remains to be normal (135–145 mmol/L), and hyponatremia correction be initiated promptly, regardless of its classification. Future work is needed to further explore the optimal spectrum of serum sodium for varied phenotypes of HF.

In our study, loop diuretics were used in 46.3%–88% patients upon admission. After hospitalization, 73%–95% HF patients were prescribed loop diuretics at discharge. Prioritizing and enhancing sodium conservation as a supplemental component of loop diuretic therapy may constitute an efficacious strategy for improving the prognosis of patients with HFcHN in the future. Tolvaptan reduced body weight and edema, and normalized serum sodium in hyponatremic patients without a significant hemodynamic change (55–57). Of note, findings from Makiko's study propose that TLV might enhance the long-term prognosis of HF patients by reducing the dosage of other diuretics or indirectly influencing the RAAS through ADH antagonism (58). Consequently, AVP antagonist administration emerges as a critical component of integrated long-term management for hyponatremia in HF cases. Adequate evidentiary support and patient-tailored dosage are needed in future research.

### Strengths and limitations

We registered our study with the Prospero Database and conducted a comprehensive systematic review and meta-analysis based on a substantial sample size of 43,316 patients. Our study adhered to the PRISMA statement guidelines. Additionally, we provided an in-depth discussion on the pathophysiology of hyponatremia in HF and conducted a thorough literature review of the available RCTs pertaining to sodium conservation treatments. However, our study also has some limitations. First, a notable degree of heterogeneity was evident among the included studies. Furthermore, all of the incorporated articles were retrospective in design, potentially introducing the influence of unrecorded variables on the observed association between serum sodium levels and outcomes. The severity and classification of the enrolled HF patients varied. Moreover, the frequency and timing of serum sodium measurements were inconsistent across the studies, as was the duration of follow-up.

## Conclusion

Our comprehensive meta-analysis underscores the substantial influence of hyponatremia on mortality across a diverse range of HF patients. Hyponatremia demonstrated significant mortality both in the short and long-term follow-up, especially in AHF or patients aged ≥50 years. Our results strongly support regular monitoring and prompt intervention for hyponatremia in the management of HF patients. In our scholarly perspective, it is recommended that sodium correction to normal spectrum be initiated promptly in cases of hyponatremia, regardless of its classification, as there is a clear linear association between serum sodium levels below 140 mmol/L and an elevated risk of mortality. Additionally, integrating newer diuretic medications like vaptans alongside traditional loop diuretics need to be explored in future clinical practice. However, further research is required to enhance the clinical understanding of its significance and determine the optimal control regimen.

## Data Availability

The original contributions presented in the study are included in the article/**Supplementary Material**, further inquiries can be directed to the corresponding author.

## References

[1] Orso F Fabbri G Maggioni AP. Epidemiology of heart failure. Handb Exp Pharmacol. (2017) 243:15–33. 10.1007/164_2016_74 27718059

[2] Vos T Flaxman AD Naghavi M Lozano R Michaud C Ezzati M Years lived with disability (YLDs) for 1160 sequelae of 289 diseases and injuries 1990–2010: a systematic analysis for the global burden of disease study 2010. Lancet. (2012) 380(9859):2163–96. 10.1016/S0140-6736(12)61729-2 23245607 PMC6350784

[3] Upadhyay A Jaber BL Madias NE. Incidence and prevalence of hyponatremia. Am J Med. (2006) 119(7 Suppl 1):S30–35. 10.1016/j.amjmed.2006.05.005 16843082

[4] Burst V. Etiology and epidemiology of hyponatremia. Front Horm Res. (2019) 52:24–35. 10.1159/000493234 32097911

[5] Rodriguez M Hernandez M Cheungpasitporn W Kashani KB Riaz I Rangaswami J Hyponatremia in heart failure: pathogenesis and management. Curr Cardiol Rev. (2019) 15(4):252–61. 10.2174/1573403X15666190306111812 30843491 PMC8142352

[6] Corona G Giuliani C Parenti G Norello D Verbalis JG Forti G Moderate hyponatremia is associated with increased risk of mortality: evidence from a meta-analysis. PLoS One. (2013) 8(12):e80451. 10.1371/journal.pone.0080451 24367479 PMC3867320

[7] Rusinaru D Tribouilloy C Berry C Richards AM Whalley GA Earle N Relationship of serum sodium concentration to mortality in a wide spectrum of heart failure patients with preserved and with reduced ejection fraction: an individual patient data meta-analysis(†): meta-analysis global group in chronic heart failure (MAGGIC). Eur J Heart Fail. (2012) 14(10):1139–46. 10.1093/eurjhf/hfs099 22782968

[8] Wang J Zhou W Yin X. Improvement of hyponatremia is associated with lower mortality risk in patients with acute decompensated heart failure: a meta-analysis of cohort studies. Heart Fail Rev. (2019) 24(2):209–17. 10.1007/s10741-018-9753-5 30535839

[9] Spasovski G Vanholder R Allolio B Annane D Ball S Bichet D Clinical practice guideline on diagnosis and treatment of hyponatraemia. Intensive Care Med. (2014) 40(3):320–31. 10.1007/s00134-014-3210-2 24562549

[10] McDonagh TA Metra M Adamo M Gardner RS Baumbach A Böhm M 2021 ESC guidelines for the diagnosis and treatment of acute and chronic heart failure. Eur Heart J. (2021) 42(36):3599–726. 10.1093/eurheartj/ehab368 34447992

[11] Stroup DF Berlin JA Morton SC Olkin I Williamson GD Rennie D Meta-analysis of observational studies in epidemiology: a proposal for reporting. Meta-analysis of observational studies in epidemiology (MOOSE) group. JAMA. (2000) 283(15):2008–12. 10.1001/jama.283.15.2008 10789670

[12] Spinar J Parenica J Vitovec J Widimsky P Linhart A Fedorco M Baseline characteristics and hospital mortality in the acute heart failure database (AHEAD) main registry. Critical Care. (2011) 15(6):R291. 10.1186/cc10584 22152228 PMC3388663

[13] Kozdag G Ertas G Emre E Akay Y Celikyurt U Sahin T Low serum triglyceride levels as predictors of cardiac death in heart failure patients. Tex Heart Inst J. (2013) 40(5):521–8. 24391311 PMC3853839

[14] Coles AH Tisminetzky M Yarzebski J Lessard D Gore JM Darling CE Magnitude of and prognostic factors associated with 1-year mortality after hospital discharge for acute decompensated heart failure based on ejection fraction findings. J Am Heart Assoc. (2015) 4(12):e002303. 10.1161/JAHA.115.002303 26702084 PMC4845282

[15] Oh C Chang HJ Sung JM Kim JY Yang W Shim J Prognostic estimation of advanced heart failure with low left ventricular ejection fraction and wide QRS interval. Korean Circ J. (2012) 42(10):659–67. 10.4070/kcj.2012.42.10.659 23170093 PMC3493802

[16] Givi M Shafie D Nouri F Garakyaraghi M Yadegarfar G Sarrafzadegan N. Survival rate and predictors of mortality in patients hospitalised with heart failure: a cohort study on the data of Persian registry of cardiovascular disease (PROVE). Postgrad Med J. (2018) 94(1112):318–24. 10.1136/postgradmedj-2018-135550 29602796

[17] Carlo CH Cardoso JN Ochia ME Oliveira MT Jr. Ramires JA Pereira-Barretto AC. Temporal variation in the prognosis and treatment of advanced heart failure - before and after 2000. Arq Bras Cardiol 2014, 102(5):495–504. 10.5935/abc.20140050 24759950 PMC4051453

[18] Carrasco-Sánchez FJ Gomez-Huelgas R Formiga F Conde-Martel A Trullàs JC Bettencourt P Association between type-2 diabetes mellitus and post-discharge outcomes in heart failure patients: findings from the RICA registry. Diabetes Res Clin Pract. (2014) 104(3):410–9. 10.1016/j.diabres.2014.03.015 24768593

[19] Herrero-Puente P Marino-Genicio R Martín-Sánchez FJ Vázquez-Alvarez J Jacob J Bermudez M Characteristics of acute heart failure in very elderly patients - EVE study (EAHFE very elderly). Eur J Intern Med. (2014) 25(5):463–70. 10.1016/j.ejim.2014.04.002 24837751

[20] Kapłon-Cieślicka A Ozierański K Balsam P Tymińska A Peller M Galas M Clinical characteristics and 1-year outcome of hyponatremic patients hospitalized for heart failure. Pol Arch Med Wewn. (2015) 125(3):120–31. 10.20452/pamw.2701 25644020

[21] Formiga F Chivite D Brasé A Petit I Moreno-Gonzalez R Arévalo-Lorido JC Clinical characteristics and prognosis in patients with a first acute heart failure hospitalization according to admission hyponatremia. Acta Clin Belg. (2018) 73(4):281–6. 10.1080/17843286.2018.1429345 29369003

[22] Felšöci M Pařenica J Spinar J Vítovec J Widimský P Linhart A Does previous hypertension affect outcome in acute heart failure? Eur J Intern Med. (2011) 22(6):591–6. 10.1016/j.ejim.2011.09.006 22075286

[23] Carrasco-Sánchez FJ Pérez-Calvo JI Morales-Rull JL Galisteo-Almeda L Páez-Rubio I Barón-Franco B Heart failure mortality according to acute variations in N-terminal pro B-type natriuretic peptide and cystatin C levels. Journal of Cardiovascular Medicine (Hagerstown, Md). (2014) 15(2):115–21. 10.2459/JCM.0b013e3283654bab 24522084

[24] Sato N Gheorghiade M Kajimoto K Munakata R Minami Y Mizuno M Hyponatremia and in-hospital mortality in patients admitted for heart failure (from the ATTEND registry). Am J Cardiol. (2013) 111(7):1019–25. 10.1016/j.amjcard.2012.12.019 23312128

[25] Bettari L Fiuzat M Shaw LK Wojdyla DM Metra M Felker GM Hyponatremia and long-term outcomes in chronic heart failure–an observational study from the duke databank for cardiovascular diseases. J Card Fail. (2012) 18(1):74–81. 10.1016/j.cardfail.2011.09.005 22196845

[26] Sato Y Yoshihisa A Oikawa M Nagai T Yoshikawa T Saito Y Hyponatremia at discharge is associated with adverse prognosis in acute heart failure syndromes with preserved ejection fraction: a report from the JASPER registry. Eur Heart J Acute Cardiovasc Care. (2019) 8(7):623–33. 10.1177/2048872618822459 30667275

[27] Vazquez R Bayes-Genis A Cygankiewicz I Pascual-Figal D Grigorian-Shamagian L Pavon R The MUSIC risk score: a simple method for predicting mortality in ambulatory patients with chronic heart failure. Eur Heart J. (2009) 30(9):1088–96. 10.1093/eurheartj/ehp032 19240065

[28] Yang M Tao L An H Liu G Tu Q Zhang H A novel nomogram to predict all-cause readmission or death risk in Chinese elderly patients with heart failure. ESC heart Failure. (2020) 7(3):1015–24. 10.1002/ehf2.12703 32319228 PMC7261546

[29] Lee DS Austin PC Rouleau JL Liu PP Naimark D Tu JV. Predicting mortality among patients hospitalized for heart failure: derivation and validation of a clinical model. Jama. (2003) 290(19):2581–7. 10.1001/jama.290.19.2581 14625335

[30] Alem MM. Predictors of mortality in patients with chronic heart failure: is hyponatremia a useful clinical biomarker? Int J Gen Med. (2020) 13:407–17. 10.2147/IJGM.S260256 32765046 PMC7381090

[31] Jafary FH Kumar M Chandna IE. Prognosis of hospitalized new-onset systolic heart failure in indo-asians–a lethal problem. J Card Fail. (2007) 13(10):855–60. 10.1016/j.cardfail.2007.07.005 18068620

[32] Yoshioka K Matsue Y Kagiyama N Yoshida K Kume T Okura H Recovery from hyponatremia in acute phase is associated with better in-hospital mortality rate in acute heart failure syndrome. J Cardiol. (2016) 67(5):406–11. 10.1016/j.jjcc.2015.12.004 26970933

[33] Rusinaru D Buiciuc O Leborgne L Slama M Massy Z Tribouilloy C. Relation of serum sodium level to long-term outcome after a first hospitalization for heart failure with preserved ejection fraction. Am J Cardiol. (2009) 103(3):405–10. 10.1016/j.amjcard.2008.09.091 19166698

[34] Kajimoto K Sato N Takano T. Relationship of renal insufficiency and clinical features or comorbidities with clinical outcome in patients hospitalised for acute heart failure syndromes. Eur Heart J Acute Cardiovasc Care. (2017) 6(8):697–708. 10.1177/2048872616658586 27363422

[35] Kajimoto K Minami Y Sato N Takano T. Serum sodium concentration, blood urea nitrogen, and outcomes in patients hospitalized for acute decompensated heart failure. Int J Cardiol. (2016) 222:195–201. 10.1016/j.ijcard.2016.07.255 27497094

[36] Senni M De Maria R Gregori D Gonzini L Gorini M Cacciatore G Temporal trends in survival and hospitalizations in outpatients with chronic systolic heart failure in 1995 and 1999. J Card Fail. (2005) 11(4):270–8. 10.1016/j.cardfail.2004.11.003 15880335

[37] Oh J Kang SM Kim IC Han S Yoo BS Choi DJ The beneficial prognostic value of hemoconcentration is negatively affected by hyponatremia in acute decompensated heart failure: data from the Korean heart failure (KorHF) registry. J Cardiol. (2017) 69(5):790–6. 10.1016/j.jjcc.2016.08.003 27590414

[38] Del Carlo CH Pereira-Barretto AC Cassaro-Strunz C Latorre Mdo R Ramires JA. Serial measure of cardiac troponin T levels for prediction of clinical events in decompensated heart failure. J Card Fail. (2004) 10(1):43–8. 10.1016/S1071-9164(03)00594-3 14966774

[39] Abebe TB Gebreyohannes EA Tefera YG Bhagavathula AS Erku DA Belachew SA The prognosis of heart failure patients: does sodium level play a significant role? PLoS One. (2018) 13(11):e0207242. 10.1371/journal.pone.0207242 30408132 PMC6224129

[40] Lee H Lee SE Park CS Park JJ Lee GY Kim M-S Hyponatraemia and its prognosis in acute heart failure is related to right ventricular dysfunction. Heart. (2018) 104(20):1670–7. 10.1136/heartjnl-2017-312084 29079633

[41] Hoorn EJ Zietse R. Diagnosis and treatment of hyponatremia: compilation of the guidelines. J Am Soc Nephrol. (2017) 28(5):1340–9. 10.1681/ASN.2016101139 28174217 PMC5407738

[42] Sterns RH Silver SM. Complications and management of hyponatremia. Curr Opin Nephrol Hypertens. (2016) 25(2):114–9. 10.1097/MNH.0000000000000200 26735146

[43] Herrera-Gómez F Monge-Donaire D Ochoa-Sangrador C Bustamante-Munguira J Alamartine E Álvarez FJ. Correction of hyponatremia may be a treatment stratification biomarker: a two-stage systematic review and meta-analysis. J Clin Med. (2018) 7(9):262. 10.3390/jcm7090262 30205538 PMC6162844

[44] De Vecchis R Di Maio M Di Biase G Ariano C. Effects of hyponatremia normalization on the short-term mortality and rehospitalizations in patients with recent acute decompensated heart failure: a retrospective study. J Clin Med. (2016) 5(10):92. 10.3390/jcm5100092 27782093 PMC5086594

[45] Sharma AK Vegh EM Kandala J Orencole M Januszkiewicz L Bose A Usefulness of hyponatremia as a predictor for adverse events in patients with heart failure receiving cardiac resynchronization therapy. Am J Cardiol. (2014) 114(1):83–7. 10.1016/j.amjcard.2014.04.009 24852916

[46] Donze JD Beeler PE Bates DW. Impact of hyponatremia correction on the risk for 30-day readmission and death in patients with congestive heart failure. Am J Med. (2016) 129(8):836–42. 10.1016/j.amjmed.2016.02.036 27019042

[47] Sorodoc V Asaftei A Puha G Ceasovschih A Lionte C Sirbu O Management of hyponatremia in heart failure: practical considerations. J Pers Med. (2023) 13(1):140. 10.3390/jpm13010140 36675801 PMC9865833

[48] Perez P C Gonzalez-Juanatey JR Nuche J Moran Fernandez L Lora Pablos D Alvarez-Garcia J Serum potassium dynamics during acute heart failure hospitalization. Clin Res Cardiol. (2022) 111(4):368–79. 10.1007/s00392-020-01753-3 33070219

[49] Ijaiya T Manohar S Lakshmi K. Therapeutic approach to the management of severe asymptomatic hyponatremia. Case Rep Nephrol. (2017) 2017:1371804. 10.1155/2017/1371804 28819575 PMC5551525

[50] Houston M. The role of magnesium in hypertension and cardiovascular disease. J Clin Hypertens (Greenwich). (2011) 13(11):843–7. 10.1111/j.1751-7176.2011.00538.x 22051430 PMC8108907

[51] Tangvoraphonkchai K Davenport A. Magnesium and cardiovascular disease. Adv Chronic Kidney Dis. (2018) 25(3):251–60. 10.1053/j.ackd.2018.02.010 29793664

[52] Price JF Kantor PF Shaddy RE Rossano JW Goldberg JF Hagan J Incidence, severity, and association with adverse outcome of hyponatremia in children hospitalized with heart failure. Am J Cardiol. (2016) 118(7):1006–10. 10.1016/j.amjcard.2016.07.014 27530824

[53] Gheorghiade M Abraham WT Albert NM Gattis Stough W Greenberg BH O'Connor CM Relationship between admission serum sodium concentration and clinical outcomes in patients hospitalized for heart failure: an analysis from the OPTIMIZE-HF registry. Eur Heart J. (2007) 28(8):980–8. 10.1093/eurheartj/ehl542 17309900

[54] Deubner N Berliner D Frey A Güder G Brenner S Fenske W Dysnatraemia in heart failure. Eur J Heart Fail. (2012) 14(10):1147–54. 10.1093/eurjhf/hfs115 22820314

[55] Gheorghiade M Niazi I Ouyang J Czerwiec F Kambayashi J Zampino M Vasopressin V2-receptor blockade with tolvaptan in patients with chronic heart failure: results from a double-blind, randomized trial. Circulation. (2003) 107(21):2690–6. 10.1161/01.CIR.0000070422.41439.04 12742979

[56] Shanmugam E Doss CR George M Jena A Rajaram M Ramaraj B Effect of tolvaptan on acute heart failure with hyponatremia–a randomized, double blind, controlled clinical trial. Indian Heart J. (2016) 68(Suppl 1):S15–21. 10.1016/j.ihj.2015.07.006 27056648 PMC4824334

[57] Konstam MA Gheorghiade M Burnett JC Jr. Grinfeld L Maggioni AP Swedberg K Effects of oral tolvaptan in patients hospitalized for worsening heart failure: the EVEREST outcome trial. JAMA. (2007) 297(12):1319–31. 10.1001/jama.297.12.1319 17384437

[58] Nakamura M Sunagawa O Kinugawa K. Tolvaptan improves prognosis in responders with acute decompensated heart failure by reducing the dose of loop diuretics. Int Heart J. (2018) 59(1):87–93. 10.1536/ihj.17-099 29375117

